# Joint Replacement Surgeries Can Be Safely Performed in HIV Patients

**DOI:** 10.3390/jcm12010293

**Published:** 2022-12-30

**Authors:** Chaofan Zhang, Yongbin Li, Yunzhi Lin, Xuehui Zhang, Zida Huang, Guochang Bai, Yao Wan, Wenming Zhang, Xinyu Fang, Wenbo Li

**Affiliations:** 1 Department of Orthopaedic Surgery, The First Affiliated Hospital, Fujian Medical University, Fuzhou 350005, China; 2 Department of Orthopaedic Surgery, National Regional Medical Center, Binhai Campus of the First Affiliated Hospital, Fujian Medical University, Fuzhou 350212, China; 3 Fujian Provincial Institute of Orthopedics, The First Affiliated Hospital, Fujian Medical University, Fuzhou 350005, China; 4 Department of Stomatology, The First Affiliated Hospital of Fujian Medical University, Fuzhou 350005, China; 5 Department of Stomatology, National Regional Medical Center, Binhai Campus of the First Affiliated Hospital, Fujian Medical University, Fuzhou 350212, China; 6 School of Health Management, Fujian Medical University, Fuzhou 350005, China; 7 Department of Orthopaedic Surgery, Dongshan Hospital, Zhangzhou 363499, China

**Keywords:** human immunodeficiency virus (HIV), highly active antiretroviral therapy (HAART), occupational exposure, joint replacement surgery

## Abstract

Whether joint replacement surgery can be performed safely on HIV patients is still a matter of debate. This study aimed to report the surgical efficacy and complications of joint replacement surgery in HIV patients. A total of 21 HIV patients and 27 non-HIV patients who underwent arthroplasties in our hospital were retrospectively reviewed. The 21 HIV patients received 29 joint replacement surgeries including 27 cases of total hip arthroplasty (THA) and 2 cases of total knee arthroplasty (TKA). The non-HIV patients received 16 THA, 10 TKA, and 3 unicompartmental arthroplasty (UKA). The length of hospital stay of HIV patients was significantly lower than that of non-HIV patients. At the last follow-up, there were no significant complications both in the HIV group and the non-HIV groups. No medical staff had any occupational exposure. We concluded that joint replacement surgery in HIV patients is safe and effective. Optimization of patients is key to treatment success. Strictly following the standardized protection protocol can prevent the risk of occupational exposure.

## 1. Introduction

Human immunodeficiency virus (HIV) infection is still a disease with high mortality and complication rates worldwide [1]. By 2018, approximately 76.1 million people in the world were HIV carriers, and 35 million people had AIDS symptoms [1]. In China, the incidence of HIV is increasing yearly. From 2009–2018, the number of HIV tests increased from 55.6 million to over 240 million, and by 2018, 861,042 people were HIV carriers [2]. The treatment of HIV has brought a heavy burden to society and the economy. With their life expectancy extended, the incidence of HIV-related comorbidities has also increased significantly, resulting in huge medical expenses [3].

The introduction of highly active antiretroviral therapy (HAART) in 1996 dramatically prolonged the survival rate of people living with HIV. HARRT therapy increases the life expectancy of HIV-positive patients to a level similar to that of the non-infected population [4]. With the extension of the life expectancy of HIV patients, their risk of combined end-stage bone and joint degenerative diseases also increases, making them candidates for orthopedic surgeries, including total hip arthroplasty (THA) and total knee arthroplasty (TKA) [5]. Meanwhile, necrosis of the femoral head (ONFH) and osteoporotic femoral neck fracture has also increased the number of THA cases among people living with HIV [6]. Many studies have demonstrated that HIV itself is an important pathological factor leading to ONFH [6]. In addition, the number of emergency orthopedic trauma surgeries conducted on HIV patients is also increasing with time [4].

A fracture usually requires emergency surgery, and for advanced joint diseases, joint replacement is the only way to relieve the pain and restore function. However, the orthopedic surgeries performed on HIV patients are different from those performed on non-HIV patients. The literature reports that the incidence of complications, primarily infection, in the HIV population is very high [7]. Patients’ impaired immune status and long-term treatment with HARRT or glucocorticoid use lead to a high risk of infection. Once an infection occurs, especially implant-related infections such as periprosthetic joint infection (PJI), the consequences are disastrous.

Whether HIV patients can be treated safely with total joint replacement or which populations are suitable for total joint replacement is still a matter of debate. To the best of our knowledge, there is little research examining this issue, especially in the Chinese population. Therefore, the primary purpose of this study was to investigate the clinical outcomes (operation time, blood loss, transfusion, hospitalization, and functional recovery) of HIV patients receiving surgery in our institution. In particular, we aim to explore the incidence of surgical site infection (SSI) in this cohort. Additionally, we also report our experience with the prevention of occupational exposure to the virus during orthopedic surgery.

## 2. Materials and Methods

### 2.1. Study Characteristics

This research protocol has been reviewed and approved by the ethics committee of our institution ([2015]084-1). Informed consent to participate in this retrospective study was obtained from all patients. The cases of HIV-infected patients admitted to our department from January 2017 to November 2022 were retrospectively reviewed. Non-HIV patients who underwent surgery on the same day were included as controls. Patients’ demographic information (age, sex, surgical side, past medical history, Harris Hip Score (HHS), perioperative laboratory tests (including white blood cell count (WBC), C-reactive protein (CRP), erythrocyte sedimentation rate (ESR), hemoglobin (Hb), albumin (ALB), CD4+ T-cell count, CD8+ T-cell count, HIV viral load (VL)), American Society of Anesthesiology (ASA) score, operation time, intraoperative blood loss, and transfusion rate) were collected; patients’ perioperative complications, hospital stay, infection occurrence rate, and the results of the last follow-up were recorded (including inflammation markers of WBC, CRP, ESR, and the HHS score).

### 2.2. Inclusion and Exclusion Criteria

#### 2.2.1. Inclusion Criteria

Patients who met the following criteria were included in this study: ① referring to the WHO diagnostic criteria for HIV [8], patients who were confirmed to be HIV carriers by blood tests; ② HIV patients who received joint replacement surgery; ③ non-HIV patients who underwent joint replacement surgery on the same day as HIV patients.

#### 2.2.2. Exclusion Criteria

Patients with the following conditions were excluded from the study: ① receiving non-arthroplasty orthopedic surgeries including open reduction and internal fixation (ORIF), ligament reconstruction, tumor removal surgery, etc.; ② debridement or revision surgery due to infection; ③ follow-up time of less than one year.

### 2.3. Surgical Technique

All operations were performed by the same experienced senior surgeon. For end-stage ONFH or femoral neck fracture, standard THA was performed. For patients with end-stage knee osteoarthritis or spontaneous osteonecrosis of the knee (SONK), TKA or unicompartmental knee arthroplasty (UKA) was performed. After the operation, rehabilitation was carried out under the guidance of physical therapists.

### 2.4. Precaution

For HIV patients, strict and standardized precautions were carried out during the whole perioperative period. The patients were admitted to a single ward. Before and after being admitted, the room was thoroughly disinfected. All medical personnel who might come into contact with the patients’ body fluids, including ward nurses, surgeons, anesthesiologists, nurses, and transporters, wore isolation gowns and protective gloves. During anesthesiology, the anesthesiologists wore a protective face shield and double surgical gloves. During surgery, the surgeons wore rubber boots and a body exhaust suit (BES) system (Meijiayisheng, Beijing, China). All steps involving sharp instruments, such as incision, hemostasis, suture, and osteotomy, were completed by the chief surgeon alone. Sharp instruments were placed in a kidney dish for transmission. After being used, the sharp instruments were immediately discarded in the sharps container. All discarded gowns, gloves, and medical waste were collected and sent for proper processing.

### 2.5. Postoperative Management

#### 2.5.1. Anticoagulation

On the first day after the operation, if there was no contradiction, the patients were routinely prescribed low molecular weight heparin (LMWH). After being discharged, the patients were usually prescribed oral rivaroxaban tablets. The dosage and duration were determined according to the American College of Chest Physicians (ACCP) guidelines [9].

#### 2.5.2. Antibiotics

Half an hour before the operation, one gram of cefazolin was administered intravenously. If the operation time was over three hours, an additional dose was added. Cefazolin was given twice every eight hours after surgery and terminated within 24 h. In cases of allergy to cefazolin, clindamycin was used.

#### 2.5.3. Rehabilitation

After the operation, the patients were asked to start rehabilitation immediately, including ankle pump exercises. For patients receiving THA and TKA, on the first day after the operation, under the guidance of occupational physical therapists, they started walking with full weight-bearing, together with other systematic rehabilitation exercises. For other orthopedic operations, if their condition allowed, functional exercises of the corresponding parts were carried out as soon as possible.

### 2.6. Outcome Measurement

The primary outcome of this study was infections leading to prosthesis removal or revision. The secondary outcomes were revisions caused by any other reason and other complications. The diagnostic criteria of PJI refer to the 2011 Musculoskeletal Infection Society (MSIS) criteria [10].

### 2.7. Statistical Analysis

The continuous data are presented as the mean ± Standard Deviation (SD), and binary data are presented as counts and percentages. An independent-sample *t*-test or Mann—Whitney U test was used for continuous values, and a paired *t*-test was used for the comparison of HHS scores before and at the last follow-up. A chi-squared test or Fisher’s exact test was used to dichotomize values according to the estimated cell size. The significance of the *p*-value was set to *p* < 0.05. Statistical analysis was performed with GraphPad Prism software (GraphPad Software, Inc., San Diego, CA, USA, V8.2.0).

## 3. Results

### 3.1. Demographics

**Figure 1:**
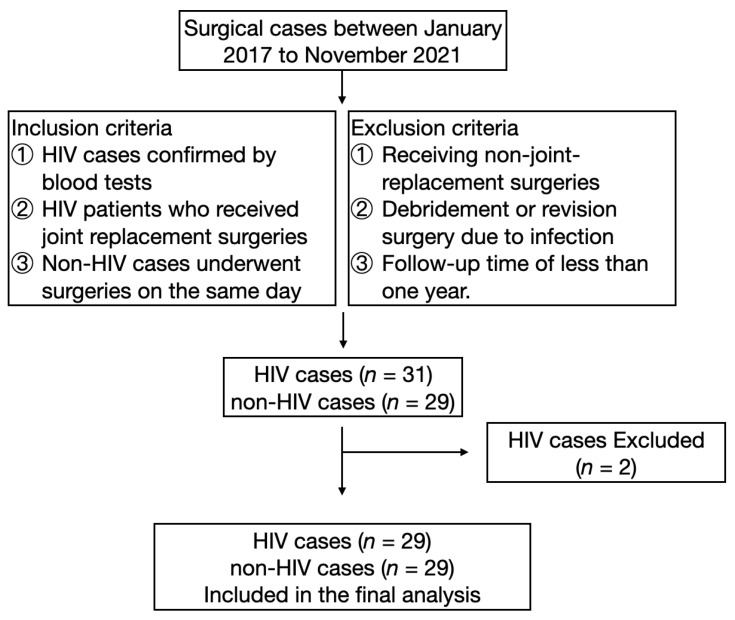
Flowchart of included and excluded patients.

From January 2017 to November 2022, a total of 23 patients with HIV were admitted to our department. Two patients whose follow-ups were less than 1 year were excluded. A flowchart of included patients is presented in Figure 1. There were 19 men and 2 women, with a mean age of 45.2 ± 11.9 years and a mean BMI of 22.9 ± 2.5. Hepatitis B virus (HBV) antigen was positive in four cases, and hepatitis C virus (HCV) antibody was positive in one case. Three patients were infected with HIV through blood transfusion, and the other patients were infected through sex. There were 19 ONFH patients and 2 femoral fracture patients who underwent THA, with 8 patients receiving bilateral THA. The preoperative HHS score was 53.1 ± 6.5. There were two knee OAs that were managed with TKA. The average CD4+ T-cell count, CD8+ T-cell count, and HIV viral load (VL) were 530.9 ± 233.8 cells/mm^3^, 860.1 ± 481.4 cells/mm^3^, and 17.9 ± 96.6 IU/mL, respectively. The patients’ demographic information is presented in Table 1.

On the same day, 29 non-HIV patients underwent joint replacement surgery. There were 12 men and 17 women, with a mean age of 65.6 ± 9.6 years and a mean BMI of 24.5 ± 3.3. Patients were comorbid with hypertension (*n* = 12), diabetes mellitus (*n* = 5), and other medical diseases. In total, 16 patients underwent THA, with no patients receiving bilateral THA. There were 10 knee OAs managed with TKA and 3 knee OAs receiving UKA.

The comparison of the relevant indicators between the two groups is shown in Table 2. The average age of the HIV group was significantly lower than that of the non-HIV group (*p* < 0.0001). The percentage of men was significantly higher in the HIV group (*p* < 0.0001). There was no significant difference in the preoperative WBC, CRP, ESR, Hb, ALB, and ASA scores between the two groups. There was no significant difference in the operative time, intraoperative blood loss, or blood transfusion rate between the two groups, but the hospitalization of HIV patients was significantly shorter than that of non-HIV patients (*p* < 0.0001).

### 3.2. Complications

After a mean follow-up of 22.9 ± 9.8 months, no complications were observed in the HIV group. Inflammatory markers, including WBC, CRP, and ESR, all returned to normal levels. The HHS score improved to 75.9 ± 6.9 (*p* < 0.0001). One case in this series (case No. 10) had repeated knee pain for more than three years and had multiple joint aspirations in an outside hospital. He was admitted with normal inflammatory markers; however, the clinical presentations and radiographic pictures showed atypical OA. Infection was considered before surgery; however, aspiration failed to draw synovial fluid from the patient. After consultation with the patient, TKA was performed, and intraoperative tissues were sent for additional culture. The cultures showed Talaromyces marneffei one week later. The patient was administered long-term antifungal treatment for one year, and the follow-up results were good. He is still under strict follow-up. After a mean follow-up of 25.4 ± 8.7 months, no complications were observed in the non-HIV group either, with WBC, CRP, and ESR all returning to normal levels.

### 3.3. Occupational Exposure

None of the medical staff had occupational exposure to HIV. No other patients were found to be infected with HIV.

## 4. Discussion

With the emergence of HARRT therapy, the survival time of HIV patients has been significantly prolonged. Their incidences of combined trauma, femoral head necrosis, and knee osteoarthritis have also increased accordingly, which usually require surgical treatment. It was reported that despite a decline in the proportion of newly reported HIV infections per year from 3.4 to 2.3 million from 2001 until 2012, approximately one-quarter of HIV-positive patients will need surgical and/or anesthetic treatment in the future [11,12]. Surgery in HIV patients has its uniqueness. First, these patients have a significantly increased risk of surgical site infection, especially in implant-related cases, due to autoimmune deficiency and long-term HARRT treatment [13]. However, with modern HARRT treatment, many HIV patients have a viral load below the measurement threshold, indicating an almost normal function of the immune system [14]. Secondly, special attention should be paid to the protection of occupational exposure when performing surgery on such patients.

Our results of 29 HIV cases receiving joint replacement surgeries showed that compared with non-HIV patients, their average age, as well as the BMI of the HIV cohort, was significantly lower. This may be because HIV is more common in young adult men. However, there was no significant difference in operation time, intraoperative blood loss, or blood transfusion rate. Nonetheless, the HIV patients had a significantly shorter hospital stay. The HIV patients underwent orthopedic surgeries with no infections, and no other complications were observed. Their follow-up results showed satisfactory pain relief and functional recovery.

The incidence of infection in HIV patients undergoing orthopedic surgery is high. Because HIV-positive patients may be immunodeficient, they are at increased risk for infection [4]. The previous literature has reported that the incidence of postoperative infection in THA patients ranges from 0% to 29%. (Table 3) A recent study reviewed 50 primary joint arthroplasty patients diagnosed with HIV including 13 TKA and 37 THA. After an average follow-up of 27 months, there were a total of 11 postoperative complications (22%), with a 5.4% infection rate for THA and a 7.7% infection rate for TKA [7]. Another study performed a retrospective database review of HIV patients who were and were not taking HAART and showed that PJI rates at 1 year were slightly higher among patients with HIV who were not taking HAART at 5.3% compared with patients with HIV who were taking HAART at 4.2% and patients without HIV at 3.8% [15]. The 2018 International Consensus Meeting on Musculoskeletal Infection held that although HIV is a risk factor for the occurrence of SSI and PJI, the risk of infection can be significantly reduced to the level of HIV-negative patients under efficient HARRT treatment [16].

Our research shows that the infection and complication rates of joint replacement surgeries in HIV patients are comparable to those in non-HIV patients and are very low. There may be a few reasons for this. First, we tried to optimize the condition of each patient before the operation, especially for patients undergoing elective surgery. The average CD4+ T-cell count, CD8+ T-cell count, and HIV viral load (VL) were 530.9 ± 233.8 cells/mm^3^, 860.1 ± 481.4 cells/mm^3^, and 17.9 ± 96.6 IU/mL, respectively, before admission. If these three indicators still suggested high HIV activity, we referred the patients to infectious disease specialists and continued antiviral treatment. HARRT should also be continued throughout the perioperative period. This is consistent with the current opinion in the literature [4]. Second, the nutritional status of patients is also important. Patients should be screened for anemia or hypoalbuminemia. Multidisciplinary cooperation among infectious disease specialists, internal medicine physicians, nutritionists, and orthopedic surgeons should be initiated to jointly optimize the preoperative nutritional and immune status of patients. Third, for HIV patients, it is particularly vital to apply the concept of enhanced recovery after surgery (ERAS) to increase the recovery of joint function and shorten the length of hospital stay. In our series, we encouraged all patients to start physical exercises as soon as possible after surgery.

In addition to infection, HIV patients are also prone to other surgery-related complications. A study by Bibas et al. reported that the risk of postoperative venous thrombosis in HIV patients was 2–10 times higher than that in non-HIV patients [17]. Our study shows that the incidence of orthopedic surgery complications in HIV patients is very low. Nonetheless, more time is needed to observe their long-term outcomes. It must also be noted that in the diagnosis and treatment of HIV patients, the possibility of infection or other comorbidities should be carefully ruled out. One patient in our series (Case 10) was initially diagnosed with knee OA and underwent TKA. Additional tests of the tissues showed a primary fungal infection of the knee (Talaromyces marneffei). Although the last follow-up of this patient was good, more time is warranted to observe the long-term outcomes.

There are many other areas for surgeons to pay attention to when operating on HIV patients, especially protection. Blood is still the main method of HIV transmission [18], and protection from HIV during the whole process of treatment is very important for a medical care provider, especially for surgeons and anesthesiologists. Henderson conducted a six-year prospective study to follow up with 1344 medical workers who were systemically exposed to HIV patients at work, including skin and mucosal contact, for an average of 30.2 months. The results showed that the risk of HIV infection through skin contact with blood was 0.3% [19]. The data in the literature also show that the risk of HIV infection after needle injury is between 0.3% and 0.03%, which is related to the needle pricking depth and blood volume [20]. Although these data show that the risk of infection after occupational exposure is not high, taking into account the seriousness of HIV infection, it is very important to apply proper protective measures. Each surgical patient should be checked for risk factors related to HIV before the operation, such as a high-risk sexual and drug abuse history, and screened with HIV blood tests. Even if the test result is negative, the possibility of a window period should be taken into account. Even for asymptomatic HIV carriers, full attention and protection should be utilized when practicing [18].

The diagnosis and treatment of HIV patients include many detailed protective measures. If available, skin staples are recommended for the suture to reduce the risk of needle injury [21]. Surgeons should wear rubber boots to avoid body fluid from contaminating the foot, and also wear a surgical helmet system and body exhaust system (‘spacesuit’ surgical gown) to avoid any contamination such as a splash to the face and naked area. This was also recommended in a recent study [22]. All the operations were performed by experienced surgeons, so as to shorten the operation time and reduce the risk of exposure. In addition to the protection during the surgical operation, the precaution should also include multidisciplinary cooperation among the department of hospital authority, operating theater, department of internal medicine, department of anesthesiology, and orthopedic ward [23].

To the best of our knowledge, this study is one of the few reports on orthopedic surgeries in the Chinese HIV-positive population. This study also has the following limitations. Firstly, this is a single-center study, which could reduce the generalizability of the results. Additionally, it involved a small number of patients, which decreased the statistical power. Secondly, we chose one year as the observation time point to investigate the incidence of infection. Infection can occur at any stage after surgery. A longer follow-up is warranted to observe the long-term treatment outcome. Thirdly, we enrolled patients receiving various kinds of arthroplasties including THA, TKA, and UKA, which would undoubtedly result in heterogeneity. The demographic and baseline characteristics of included patients may also appear homogenous. Fourthly, sex imbalance was inevitable because the HIV-positive patients in this study were mostly male patients, while the difference in the non-HIV group was non-significant. Lastly, because of the retrospective nature of the study, it was not possible to control for other factors that impact the surgical outcomes. Despite these limitations, this study is unique and provides the foundation for future studies with regard to patients with HIV receiving joint replacement surgeries.

**Table 3:** THA surgeries performed in HIV patients as reported in the literature.

Study	HIV Cases	Diagnosis	TJAs	HARRT	CD4+ T Cells	HIV VL	FU	Revision	PJI
Jacob et al. [7]	50	N/A	13 TKA, 37 THA	All	683	<20 in 95% of patients	27 months	THA 8.1%, TKA 27.2%	5.4% THA, 7.7% TKA
Olson et al. [24]	110	65 OA, 37 ONFH, 5 Fracture,	85 THA, 25 TKA	N/A	581	17	5.5 years	N/A	N/A
Delanois et al. [15]	1202	N/A	THA	601	N/A	N/A	N/A	3.3%	4.7%
Roof et al. [25]	25	24 OA, 1 Traumatic arthritis	25 TKA		815.86	2827.62	18.8 months	1 (4%)	1 (4%)
Novikov et al. [5]	27	N/A	36	All	444	N/A	78.9 months	5 (13.9%)	1 (2.8%)
Mahure et al. [26]	638	389 OA, 249 ONFH	THA	N/A	N/A	N/A	N/A	8 (1.6%)	1 (0.2%)
Chang et al. [27]	16	ONFH	25 THA	N/A	464.1	All undetectable	64.6 months	0	0

Abbreviations: TJA: total joint arthroplasty; THA: total hip arthroplasty; TKA: total knee arthroplasty; HARRT: highly active antiretroviral therapy; VL: viral load; FU: follow-up; PJI: Periprosthetic joint infection; OA: osteoarthritis; ONFH: osteonecrosis of femoral head; N/A: not available.

## 5. Conclusions

We concluded from the current study that joint replacement surgery in HIV patients is safe and effective. Optimization of patients is key to treatment success. Strictly following a standardized protection protocol can reduce the risk of occupational exposure.

## Figures and Tables

**Table 1:** Demographics of 21 HIV patients undergoing 29 joint replacement surgeries.

Case	Sex/Age	Side	Diagnosis	Past History	HARRT	CD4+ T Cells (Cells/mm^3^)	CD8+ T Cells (Cells/mm^3^)	HIV Viral Load (IU/mL)	Surgery	Operation Time (Min)	Hospitalization (Days)	Complications
1	M/38	R	ONFH	HBV carrier	Yes	543	1129	Undetected	THA	75	3	None
2	M/38	L	ONFH	HBV carrier	Yes	543	1129	Undetected	THA	105	3	None
3	F/57	L	OA	Atrial fibrillation	Yes	325	682	Undetected	TKA	210	8	None
4	M/34	R	ONFH	None	Yes	337	1789	Undetected	THA	80	5	None
5	M/34	L	ONFH	None	Yes	337	1789	Undetected	THA	70	5	None
6	M/54	L	Femoral neck fracture	None	Yes	422	1439	520	THA	90	6	None
7	M/55	R	ONFH	None	Yes	495	307	Undetected	THA	80	7	None
8	M/63	R	ONFH	None	NO	333	1618	Undetected	THA	135	2	None
9	F/50	L	ONFH	None	Yes	461	569	Undetected	THA	120	2	None
10	M/45	R	OA	HBV carrier	Yes	1170	330	Undetected	TKA	158	6	None
11	M/63	L	ONFH	None	NO	435	553	Undetected	THA	90	2	None
12	M/63	R	ONFH	None	NO	435	553	Undetected	THA	75	2	None
13	M/79	L	Intertrochanteric fracture	HT	Yes	476	65	Undetected	THA	150	3	None
14	M/46	R	ONFH	HBV carrier	Yes	340	547	Undetected	THA	95	2	None
15	M/31	R	ONFH	None	Yes	745	688	Undetected	THA	75	2	None
16	M/31	L	ONFH	None	Yes	745	688	Undetected	THA	90	2	None
17	M/46	L	ONFH	None	NO	402	1090	Undetected	THA	70	2	None
18	M/28	R	ONFH	None	Yes	468	887	Undetected	THA	70	2	None
19	M/28	L	ONFH	None	Yes	468	887	Undetected	THA	70	2	None
20	M/40	L	ONFH	None	Yes	180	450	Undetected	THA	90	3	None
21	M/40	R	ONFH	None	Yes	180	450	Undetected	THA	80	3	None
22	M/39	R	ONFH	None	Yes	450	498	Undetected	THA	95	3	None
23	M/36	L	ONFH	None	Yes	500	548	Undetected	THA	100	3	None
24	M/46	L	ONFH	None	Yes	550	632	Undetected	THA	90	3	None
25	M/44	L	ONFH	None	Yes	1002	1647	Undetected	THA	70	3	None
26	M/44	R	ONFH	None	Yes	1002	1647	Undetected	THA	75	3	None
27	M/48	R	ONFH	HT	Yes	711	905	Undetected	THA	70	3	None
28	M/48	L	ONFH	HT	Yes	711	905	Undetected	THA	75	3	None
29	M/42	L	ONFH	None	Yes	629	521	Undetected	THA	76	3	None

Abbreviations: R: right; L: left; M: male; F: female; ONFH: osteonecrosis of the femoral head; OA: osteoarthritis; HBV: hepatitis B virus; HT: hypertension; HARRT: highly active antiretroviral therapy; HIV: human immunodeficiency virus; THA: total hip arthroplasty; TKA: total knee arthroplasty).

**Table 2:** Statistical analysis of perioperative indicators between HIV patients and non-HIV patients.

	HIV Cases	Non-HIV Controls	*p*-Value
Number	29	29	-
Age	45.2 ± 11.9	65.6 ± 9.6	<0.0001
Sex	27:2	12:17	<0.0001
BMI	22.9 ± 2.5	24.5 ± 3.3	0.06
HT	3	12	0.007
DM	0	5	0.02
Hepatitis B	4	1	0.16
Hepatitis C	1	0	0.31
WBC (×10^9^/L)	6.2 ± 1.5	6.9 ± 1.9	0.13
CRP (mg/L)	7.0 ± 4.6	13.8 ± 33.8	0.33
ESR (mm/h)	24.7 ± 17.8	29.9 ± 17.2	0.27
Hb (g/L)	145.0 ± 8.0	138.0 ± 17.9	0.07
ALB (g/L)	44.2 ± 4.0	43.2 ± 3.2	0.28
ASA score	1.6 ± 0.7	1.9 ± 0.5	0.06
Operation time (min)	94.8 ± 33.5	109.8 ± 25.3	0.06
Blood loss (mL)	151.2 ± 98.3	166.6 ± 92.5	0.55
Transfusion	1/29	1/29	>0.99
Hospitalization (days)	3.3 ± 1.7	6.8 ± 3.5	<0.0001
Infection rate	0/29	0/29	>0.99
Follow-up time (months)	22.9 ± 9.8	25.4 ± 8.7	0.31

(abbreviations: BMI: body mass index; HT: hypertension; DM: diabetes mellitus; WBC: white blood cell count; CRP: C-reactive protein; ESR: erythrocyte sedimentation rate; Hb: hemoglobin; ALB: albumin; ASA: American Society of Anesthesiology).

## Data Availability

The data are available upon request from the corresponding author.

## References

[1] Girum T., Wasie A., Worku A. (2018). Trend of HIV/AIDS for the Last 26 Years and Predicting Achievement of the 90-90-90 HIV Prevention Targets by 2020 in Ethiopia: A Time Series Analysis. BMC Infect. Dis..

[2] Ding Y., Ma Z., He J., Xu X., Qiao S., Xu L., Shi R., Xu X., Zhu B., Li J. (2019). Evolving HIV Epidemiology in Mainland China: 2009–2018. Curr. HIV/AIDS Rep..

[3] Zhuang X., Chen Y., Wu Z., Scott S.R., Lu R., Xu Z., Yu Y., Wang W., Cao L., Liang Y. (2020). Analysis of Hospitalization Expenses of 610 HIV/AIDS Patients in Nantong, China. BMC Health Serv. Res..

[4] Grabowski G., Pilato A., Clark C., Jackson J.B. (2017). HIV in Orthopaedic Surgery. J. Am. Acad. Orthop. Surg..

[5] Novikov D., Anoushiravani A.A., Chen K.K., Wolfson T.S., Snir N., Schwarzkopf R. (2019). Total Hip Arthroplasty in Human Immunodeficiency Virus–Positive Patients: A Concise Follow-Up at 10 to 14 Years. J. Arthroplast..

[6] Maharaj Z. (2020). Human Immunodeficiency Virus in Total Hip Arthroplasty. EFORT Open Rev..

[7] Jacob R., Chandler K., Medawar N., Sowers M., Mcgwin G., Naranje S. (2022). Incidence of Complications and Revision Surgery in HAART Compliant HIV Patients Undergoing Primary Total Hip and Knee Arthroplasty: An Institutional Review. Arch. Orthop. Trauma Surg..

[8] World Health Organization (2016). Clinical Guidelines: HIV Diagnosis. Consolidated Guidelines on the Use of Antiretroviral Drugs for Treating and Preventing HIV Infection: Recommendations for a Public Health Approach.

[9] Falck-Ytter Y., Francis C.W., Johanson N.A., Curley C., Dahl O.E., Schulman S., Ortel T.L., Pauker S.G., Colwell C.W. (2012). Prevention of VTE in Orthopedic Surgery Patients. Antithrombotic Therapy and Prevention of Thrombosis: American College of Chest Physicians Evidence-Based Clinical Practice Guidelines. Chest.

[10] Parvizi J., Zmistowski B., Berbari E.F., Bauer T.W., Springer B.D., Della Valle C.J., Garvin K.L., Mont M.A., Wongworawat M.D., Zalavras C.G. (2011). New Definition for Periprosthetic Joint Infection. From the Workgroup of the Musculoskeletal Infection Society. Clin. Orthop. Relat. Res..

[11] (2013). Global Report 2013—UNAids Report on the Global Epidemic.

[12] Prout J., Agarwal B. (2005). Anaesthesia and Critical Care for Patients with HIV Infection. Contin. Educ. Anaesth. Crit. Care Pain.

[13] O’Neill S.C., Queally J.M., Hickey A., Mulhall K.J. (2019). Outcome of Total Hip and Knee Arthroplasty in HIV-Infected Patients: A Systematic Review. Orthop. Rev..

[14] Di Y., Zhao X.Y., Ye J.J., Li B., Ma N. (2019). Fundus Manifestations and HIV Viral Loads of AIDS Patients before and after HAART. Int. J. Ophthalmol..

[15] Delanois R.E. (2021). Complication Rates after Total Hip Arthroplasty. JBJS Open Access.

[16] Enayatollahi M.A., Mokete L., Sanchez M., Pietrzak J.R.T. QUESTION: Does Human Immunodeficiency Virus (HIV) Predispose Patients to Surgical Site Infection/Periprosthetic Joint Infection (SSI/PJI)? If so, What Optimization Should Be Undertaken Prior to Operating on Patients with HIV?. Proceedings of the Second International Consensus Meeting on Musculoskeletal Infection.

[17] Bibas M., Biava G., Antinori A. (2011). HIV-Associated Venous Thromboembolism. Mediterr. J. Hematol. Infect. Dis..

[18] Grzela TWyżgowski P., Rosiek A., Grzela T., Leksowski K. (2016). Occupational HIV Risk for Health Care Workers: Risk Factor and the Risk of Infection in the Course of Professional Activities. Ther. Clin. Risk Manag..

[19] Henderson D.K., Fahey B.J., Willy M., Schmitt J.M., Carey K., Koziol D.E., Lane H.C., Fedio J., Saah A.J. (1990). Risk for Occupational Transmission of Human Immunodeficiency Virus Type 1 (HIV-1) Associated with Clinical Exposures: A Prospective Evaluation. Ann. Intern. Med..

[20] Wilson S. (2009). HIV and Anaesthesia. Updat. Anaesth..

[21] Ray R., Chatterjee S. (2021). Review Article Institutional Guidelines for Safe Surgery in HIV Patients in a Government Medical College. J. Indian Med. Assoc..

[22] Makovicka J.L., Bingham J.S., Patel K.A., Young S.W., Beauchamp C.P., Spangehl M.J. (2018). Surgeon Personal Protection:An Underappreciated Benefit of Positive-Pressure Exhaust Suits. Clin. Orthop. Relat. Res..

[23] Wiznia D.H., Morgan R.C., Gibson D. (2022). Movement Is Life—Optimizing Patient Access to Total Joint Arthroplasty: HIV and Hepatitis C Disparities. J. Am. Acad. Orthop. Surg..

[24] Olson J.J., Jackson J., Lange J.K., Bedair H.S. (2021). HIV-Positive Patients Are at Increased Risk of Venous Thromboembolism after Total Joint Replacement. JAAOS—J. Am. Acad. Orthop. Surg..

[25] Roof M.A., Anoushiravani A.A., Chen K.K., Moses M.J., Wolfson T., Poultsides L., Schwarzkopf R. (2020). Outcomes of Total Knee Arthroplasty in Human Immunodeficiency Virus-Positive Patients. J. Knee Surg..

[26] Mahure S.A., Bosco J.A., Slover J.D., Vigdorchik J., Iorio R., Schwarzkopf R. (2018). Risk of Complications after THA Increases among Patients Who Are Coinfected with HIV and Hepatitis C. Clin. Orthop. Relat. Res..

[27] Chang C., Tsai S., Chen C., Wu P. (2018). Optimal Timing for Elective Total Hip Replacement in HIV-Positive Patients. Orthop. Traumatol. Surg. Res..

